# Fixed-Time Coverage Control of Mobile Robot Networks Considering the Time Cost Metric

**DOI:** 10.3390/s22228938

**Published:** 2022-11-18

**Authors:** Qihai Sun, Tianjun Liao, Zhi-Wei Liu, Ming Chi, Dingxin He

**Affiliations:** 1 School of Artificial Intelligence and Automation, Huazhong University of Science and Technology, Wuhan 430074, China; 2 Academy of Military Sciences, Beijing 100000, China

**Keywords:** multi-robot network, dynamic environment coverage, time cost metric, fixed-time control

## Abstract

In this work, we studied the area coverage control problem (ACCP) based on the time cost metric of a robot network with an input disturbance in a dynamic environment, which was modeled by a time-varying risk density function. A coverage control method based on the time cost metric was proposed. The area coverage task that considers the time cost consists of two phases: the robot network is driven to cover the task area with a time-optimal effect in the first phase; the second phase is when the accident occurs and the robot is driven to the accident site at maximum speed. Considering that there were movable objects in the task area, a time-varying risk density function was used to describe the risk degree at different locations in the task area. In the presence of the input disturbance, a robust controller was designed to drive each robot, with different maximum control input values, to the position that locally minimized the time cost metric function in a fixed time, and the conditions for maximum control input were obtained. Finally, simulation results and comparison result are presented in this paper.

## 1. Introduction

Multi-robot cooperative control has drawn increasing attention from academics throughout the world due to the ongoing development of robotics technologies and related theories [1,2,3,4]. Robots need to be effectively controlled in various applications [5]. The ACCP is a crucial area of research in multi-robot cooperative control because it deals with how the robot network is distributed spatially within the area of interest so that it can be successfully observed or sensed [6,7,8]. The division of the task area into many sub-areas, with each robot solely covering its respective sub-area, is a key tactic for the area coverage of a multi-robot network. Researchers have frequently exploited the divide-and-conquer strategy to create area coverage control techniques, the most notable of which being Voronoi partitioning [9].

Based on the Voronoi partition method, some work has considered the ACCP in different environments and application scenarios. The authors of [10] proposed a cooperative area exploration strategy of the robot network based on the Voronoi partitioning approach. Based on the Voronoi partition method, the deployment of unmanned aerial vehicles (UAVs), while maintaining the connection between UAVs and base stations, was studied in [11]. Robots with different sensing abilities performed area coverage tasks in [12,13]. Coverage control was carried out for mobile robots with limited sensing or communication in distance [14,15,16]. An adaptive method was proposed to deploy sensor nodes to sense an area with unknown environmental density [17]. The non-parametric Gaussian statistical regression method to estimate density function online was used in [18].

Generally, the environmental density function, which describes the importance of each position in the task area, is time-invariant. However, in some applications, there may be some important movable objects in the task area, and their influence on the area environment dynamically changes. Therefore, the environment density functions in some practical application scenarios are time-varying functions. Consider that there are movable objects in an ACCP, ref. [19] proposed a decentralized control law for the mobile robot network. Neutralization of pollutants in a area with mobile pollution sources was addressed in [20]. In the dynamic environment, the discrete coverage control problem was solved based on the k-means method in [21]. Considering unicyle model robots, the area coverage control of underactuated robots in dynamic environments was studied in [22,23].

The purpose of the above research about ACCPs is to minimize the sensing cost or maximize the monitoring probability. However, if the goal is to respond quickly to accidents in the area, the coverage control above will no longer be applicable, and the time cost should be used to measure the coverage effect. A few of scholars have paid attention to the time cost of coverage and have proposed some time-optimized coverage control (T-OCC) methods. Ru et al., considering both energy loss and moving time, ref. [24] solved the minimum cost coverage control problem by using a multi-objective optimization function. When there is a drift field, the ACCP considering time cost for the unicycle network was solved in [25]. For time-sensitive coverage tasks, ref. [26] solved the ACCP when the maximum velocity of each robot was different. The ACCP based on time cost is generally divided into two phases. The time cost is considered in the metric function of the coverage effect, and the robot network is driven to minimize the time cost metric function (TCMF) in the first phase. The second phase is when an accident occurs and the robot is driven to reach the accident site at maximum speed. However, in the current study of T-OCC [24,25,26], the first phase was achieved asymptotically, which did not ensure a quick response of the robot network.

Unlike other works that aimed to maximize the coverage monitoring probability or minimize the sensing cost in ACCP, the influence of the coverage time cost was considered, and the ACCP was solved with respect to the robot network in a dynamic environment. In addition, as opposed to the existing work related to the ACCP, a fixed-time robust controller was designed to drive each robot with a different maximum control input to minimize the TCMF, considering input disturbances, and the conditions that the control input should satisfy were analyzed. Finally, several simulation results were obtained, and the results of the comparison between the proposed control and the classical Lloyd algorithm [9,26] are presented in this paper.

This remainder of this paper is organized as follows. The kinematics of the robot and the generalized Voronoi partition method based on the shortest time principle are presented in Section 2. The coverage metric function with respect to time cost is given and analyzed in Section 3. The control law of time optimal coverage effect is presented in Section 4. Section 5 and Section 6 present, respectively, the simulation results and conclusions.

## 2. Preliminaries

Mobile robots mainly include aerial aircraft [27,28], mobile cars on the road [29,30,31] and unmanned surface vessels on the water [32,33]. If we consider that *N* single integral model robots are used in a two-dimensional convex task area $Q\in R^{2}$, we define the robots’ set V ($V=\left\{1,2,\ldots ,N\right\}$), and the robots’ kinematics are modeled as [29,34]

(1) $$\dot{p}=\left[\begin{array}{c} \dot{x}\\ \dot{y} \end{array}\right]=\left[\begin{array}{c} u_{x}\\ u_{y} \end{array}\right]+\left[\begin{array}{c} d_{x}\\ d_{y} \end{array}\right]=u+d,$$

where $p={\left[x,y\right]}^{T}\in R^{2}$ is a robot’s position in the earth-fixed frame, the control input is $u={\left[u_{x},u_{y}\right]}^{T}$ and the disturbance $d={\left[d_{x},d_{y}\right]}^{T}$ is bounded (‖d(t)‖<ρ). It is assumed that the maximum control inputs of each robot are different and bounded.

**Figure 1:**
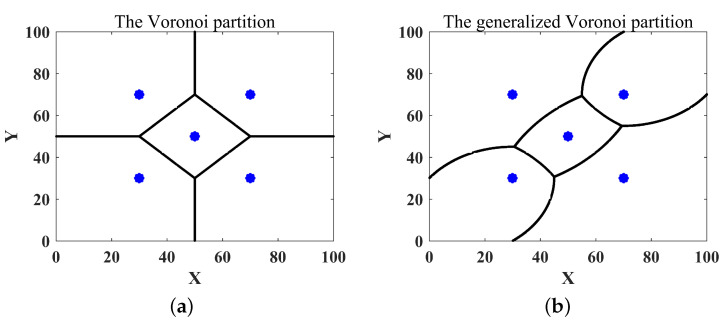
The Voronoi partition based on the proximity principle (**a**) and the generalized Voronoi partition based on the shortest time principle (**b**) (where the blue point “•” represents the position of the robot, and each robot had a different maximum speed ${v_{i\_}}_{\max}\left(i\in V\right)$).

This paper considered coverage control with respect to the time cost metric. Inspired by Voronoi division [9,35], which is based on the principle of proximity as shown in Figure 1a, the task area *Q* was divided using the principle of shortest time, called the generalized Voronoi partition, as shown in Figure 1b:

(2) $$V_{i}=\left\{q\in Q|t_{\left(p_{i},q\right)}\leq t_{\left(p_{j},q\right)},\forall j\in V\right\},$$

where $t_{\left(p_{i},q\right)}=\frac{\|p_{i}-q\|}{{v_{i\_}}_{\max}}$ is the minimum time taken for the *i*-th robot to move from position $p_{i}$ to position *q*(q∈Q) at the maximum speed ${v_{i\_}}_{\max}$.

The risk degree in *Q* is described by a time-varying function ψ(q,t),

$$\psi \left(q,t\right)=\phi \left(q\right)+\sum_{j=1}^{M}\phi_{j}\left(q,t\right),$$

where ϕ(q) represents the constant risk in the area and $\phi_{j}\left(q,t\right)$(j=1,…,M) is the contribution of the *j*-th movable object on the task area. This was different from most studies that have only considered the time-invariant risk ϕ(q) [7,8,9,12,13,14,15,16,17,18,24,25,26].

## 3. Time Optimal Coverage Analysis

The purpose of this paper was to dynamically deploy the robot network in the task area to achieve the time optimal coverage effect, which was quantified by the TCMF

(3) $$H_{t}\left(P,t\right)=\sum_{i=1}^{N}\int_{V_{i}}{\left(t_{\left(p_{i},q\right)}\right)}^{2}\psi \left(q,t\right)dq.$$

**Lemma** **1** (The Leibniz integral rule [36]). *The area V, which is smoothly dependent on position p, has a uniquely defined outer normal vector n(q) anywhere on its boundary ∂V(q). For the function*

$$\Omega =\int_{V_{\left(q\right)}}h(p,q)dq,$$

*one has*

$$\frac{\partial \Omega }{\partial p}=\int_{V_{\left(q\right)}}\frac{\partial h(p,q)}{\partial p}dq+\int_{\partial V_{\left(q\right)}}h\left(p,q\right)n{\left(q\right)}^{T}\frac{\partial q}{\partial p}dq.$$

For the TCMF (3), one has

(4) $$\begin{array}{c} \begin{array}{cc} H_{t}\left(P,t\right)= & \int_{V_{i}}{\left(t_{\left(p_{i},q\right)}\right)}^{2}\psi \left(q,t\right)dq\\ \; & +\sum_{j\in N_{i}}\int_{V_{j}}{\left(t_{\left(p_{i},q\right)}\right)}^{2}\psi \left(q,t\right)dq\\ \; & +\sum_{j\notin \left\{i\right\}\cup N_{i}}\int_{V_{j}}{\left(t_{\left(p_{i},q\right)}\right)}^{2}\psi \left(q,t\right)dq, \end{array} \end{array}$$

where $N_{i}$ is the neighbor set of the robot *i*, which is defined as the other robots that the Voronoi partitions have a common edge, $V_{i}$, with. The $p_{i}$ partial derivative of $H_{t}\left(P,t\right)$ yields

(5) $$\begin{array}{c} \begin{array}{cc} \frac{\partial H_{t}\left(P,t\right)}{\partial p_{i}}= & \frac{\partial \left(\int_{V_{i}}{\left(t_{\left(p_{i},q\right)}\right)}^{2}\psi \left(q,t\right)dq\right)}{\partial p_{i}}\\ \; & +\frac{\partial \left(\sum_{j\in N_{i}}\int_{V_{j}}{\left(t_{\left(p_{i},q\right)}\right)}^{2}\psi \left(q,t\right)dq\right)}{\partial p_{i}}. \end{array} \end{array}$$

According to the Leibniz formula, one has the following:

(6) $$\begin{array}{c} \begin{array}{cc} \frac{\partial H_{t}\left(P,t\right)}{\partial p_{i}} & \\ = & \frac{2}{v_{i\_}{{}_{\max}}^{2}}\int_{V_{i}}{\left(p_{i}-q\right)}^{T}\psi \left(q,t\right)dq\\ \; & +\sum_{j\in N_{i}}\int_{\partial V_{i,j}}{\left(\frac{\|p_{i}-q\|}{{v_{i\_}}_{\max}}\right)}^{2}\psi \left(q,t\right)n_{i}{\left(q\right)}^{T}\frac{\partial q}{\partial p}dq\\ \; & +\sum_{j\in N_{i}}\int_{\partial V_{j}}{\left(\frac{\|p_{j}-q\|}{{v_{j\_}}_{\max}}\right)}^{2}\psi \left(q,t\right)n_{j}{\left(q\right)}^{T}\frac{\partial q}{\partial p}dq, \end{array} \end{array}$$

where $\partial V_{i,j}$ is the common edge of $V_{i}$ and $V_{j}$ and $n_{i}\left(q\right),n_{j}\left(q\right)$ are the outward normal vectors of $V_{i},V_{j}$ at the boundary $\partial V_{i,j}$, respectively, and one has

(7) $$n_{i}\left(q\right)=-n_{j}\left(q\right).$$

Substituting (7) into Equation (6) yields

(8) $$\begin{array}{c} \begin{array}{cc} \; & \frac{\partial H_{t}\left(P,t\right)}{\partial p_{i}}\\ = & \frac{2}{v_{i\_}{{}_{\max}}^{2}}\int_{V_{i}}{\left(p_{i}-q\right)}^{T}\psi \left(q,t\right)dq+\sum_{j\in N_{i}}\int_{\partial V_{i,j}}{\left(\frac{\|p_{i}-q\|}{{v_{i\_}}_{\max}}\right)}^{2}\psi \left(q,t\right)n_{i}\left(q\right)\frac{\partial q}{\partial p}dq\\ \; & -\sum_{j\in N_{i}}\int_{\partial V_{i,j}}{\left(\frac{\|p_{j}-q\|}{{v_{j\_}}_{\max}}\right)}^{2}\psi \left(q,t\right)n_{i}\left(q\right)\frac{\partial q}{\partial p}dq\\ = & \frac{2}{v_{i\_}{{}_{\max}}^{2}}\int_{V_{i}}{\left(p_{i}-q\right)}^{T}\psi \left(q,t\right)dq\\ \; & +\sum_{j\in N_{i}}\int_{\partial V_{i,j}}\left[{\left(\frac{\|p_{i}-q\|}{{v_{i\_}}_{\max}}\right)}^{2}-{\left(\frac{\|p_{j}-q\|}{{v_{j\_}}_{\max}}\right)}^{2}\right]\psi \left(q,t\right)n_{i}\left(q\right)\frac{\partial q}{\partial p}dq. \end{array} \end{array}$$

Note that, when the point *p* is on the common edge $\partial V_{i,j}$, one has

(9) $${t_{\min}}_{\left(p_{i},q\right)}={t_{\min}}_{\left(p_{j},q\right)}\Rightarrow \frac{\|p_{i}-q\|}{{v_{i\_}}_{\max}}=\frac{\|p_{j}-q\|}{{v_{j\_}}_{\max}}.$$

Substituting (9) into (8) yields

(10) $$\begin{array}{c} \begin{array}{cc} \frac{\partial H_{t}\left(P\right)}{\partial p_{i}}= & \frac{2}{v_{i\_}{{}_{\max}}^{2}}\int_{V_{i}}{\left(p_{i}-q\right)}^{T}\psi \left(q,t\right)dq\\ = & \frac{2}{v_{i\_}{{}_{\max}}^{2}}M_{V_{i}}\left(p_{i}-C_{V_{i}}\right), \end{array} \end{array}$$

where

$$M_{i}=\int_{V_{i}}\psi (q,t)dq$$

is the mass of $V_{i}$, and

$$C_{i}=\left[\begin{array}{c} C_{xi}\\ C_{yi} \end{array}\right]=\frac{\int_{V_{i}}q\psi (q,t)dq}{\int_{V_{i}}\psi (q,t)dq}$$

is the centroid of $V_{i}$.

Obviously, if the position $p_{i}$ coincides with the centroid $C_{i}$, the $p_{i}$ derivative of metric function $H_{t}\left(P,t\right)$ is zero. That is, the robot *i* achieves the optimal coverage effect of the partition $V_{i}$ with the metric function $H_{t}\left(P,t\right)$. When each robot achieves the optimal coverage effect of its Voronoi partition, the robot network achieves the local optimal coverage effect of the task area *Q*. Next, the T-OCC of the robot network was designed to achieve a time-optimal coverage effect.

## 4. Fixed-Time Coverage Control

**Figure 2:**
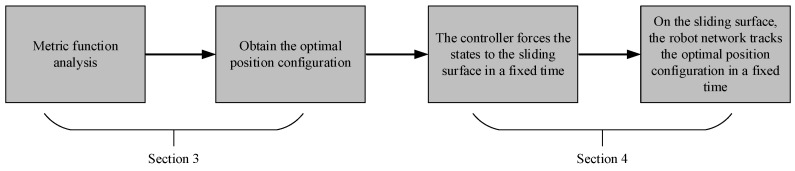
Fixed-time coverage control process of a mobile robot network with respect to the time cost metric.

The above chapter analyzed and obtained the optimal position configuration of a robot network. Based on the sliding mode control method and the fixed-time stability theory, the fixed-time coverage controller was designed for a robot network. First, the controller forced states in the robot network to stabilize it on the sliding surfaces in a fixed time. Then, the position configuration of the robot network could track the optimal position configuration on the sliding surface in a fixed time. The control process is shown in Figure 2.

**Lemma** **2** (Fixed-time stability theory [37,38,39]). *Consider the system $\dot{x}\left(t\right)=f\left(x\left(t\right)\right)$, if there is a function $V\left(x\right):R^{N}\to R^{N}$ that is continuously positive definite and there are real numbers $k_{1},k_{2}>0$, q>1, p∈(0,1) that satisfy:*

$$\dot{V}\left(x\right)\leq -\left(k_{1}{\left(V\left(x\right)\right)}^{q}+k_{2}{\left(V\left(x\right)\right)}^{p}\right),x\in R^{N}\backslash \left\{0\right\},$$

*then the system can stabilize to the origin in fixed time.*

Define the position error as follows:

$$e_{i}=\left[\begin{array}{c} e_{xi}\\ e_{yi} \end{array}\right]=\left[\begin{array}{c} x_{i}-C_{xi}\\ y_{i}-C_{yi} \end{array}\right]\left(i\in V\right).$$

Design the sliding surfaces as follows:

(11) $$\left[\begin{array}{c} {s_{x}}_{i}\\ {s_{y}}_{i} \end{array}\right]=\left[\begin{array}{c} x_{i}-{g_{x}}_{i}\\ y_{i}-{g_{y}}_{i} \end{array}\right]\left(i\in V\right).$$

The variables ${g_{x}}_{i}$ and ${g_{y}}_{i}$ are defined as follows:

(12) $$\begin{array}{c} {\dot{g}}_{xi}={\dot{C}}_{xi}-k_{1}\left\|e_{xi}{\|}^{\alpha_{1}}sgn\left(e_{xi}\right)-k_{2}\right\|e_{xi}{\|}^{\alpha_{2}}sgn\left(e_{xi}\right),\\ {\dot{g}}_{yi}={\dot{C}}_{yi}-k_{3}\left\|e_{yi}{\|}^{\alpha_{3}}sgn\left(e_{yi}\right)-k_{4}\right\|e_{yi}{\|}^{\alpha_{4}}sgn\left(e_{yi}\right), \end{array}$$

where $k_{1},k_{2},k_{3},k_{4}$ are positive constant coefficients, $0<\alpha_{1},\alpha_{3}<1$, $\alpha_{2},\alpha_{4}>1$ and ${\dot{C}}_{i}$ is a bounded time derivative [40].

Considering the presence of the input disturbance, the fixed-time controller for the robot *i* can be designed as

(13) $$u_{i}=\left[\begin{array}{c} u_{xi}\\ u_{yi} \end{array}\right]=\left[\begin{array}{c} {\dot{g}}_{xi}-b_{1}\|s_{xi}{\|}^{\beta_{1}}sgn\left(s_{xi}\right)\\ -b_{2}\|s_{xi}{\|}^{\beta_{2}}sgn\left(s_{xi}\right)-\rho \cdot sgn\left(s_{xi}\right)\\ {\dot{g}}_{yi}-b_{3}\|s_{yi}{\|}^{\beta_{3}}sgn\left(s_{yi}\right)\\ -b_{4}\|s_{yi}{\|}^{\beta_{4}}sgn\left(s_{yi}\right)-\rho \cdot sgn\left(s_{yi}\right) \end{array}\right],$$

where the coefficients $b_{1},b_{2},b_{3},b_{4}>0$, $-1<\beta_{1},\beta_{3}<0$ and $\beta_{2},\beta_{4}>1$.

Let

$${u_{xi\_}}_{\max}=\max(||u_{xi}\left(t\right)||),{u_{yi\_}}_{\max}=\max(||u_{yi}\left(t\right)||)$$

be the maximum values of the control input $u_{xi},u_{yi}$, the main result is stated as follows.

**Theorem** **1.** *For the mobile robot network with dynamics (1), the controller (13) can drive the state of robot i(i∈V) to reach the sliding surface (11) within the fixed time $T_{1}$, and the time $T_{1}$ depends only on the controller parameters, not on the initial state of the robot i,*

$$T_{1}=\frac{1}{2{\left(b_{1}\right)}^{\frac{2}{\beta_{1}+1}}\left(1-\frac{\beta_{1}+1}{2}\right)}+\frac{1}{2{\left(b_{2}\right)}^{-\frac{2}{\beta_{2}+1}}\left(\frac{\beta_{2}+1}{2}-1\right)},$$

*where the maximum values of the control inputs need to be*

$$\left[\begin{array}{c} {u_{xi\_}}_{\max}\\ {u_{yi\_}}_{\max} \end{array}\right]\geq \left[\begin{array}{c} \rho +\|{\dot{g}}_{xi}\|\\ \rho +\|{\dot{g}}_{xi}\| \end{array}\right].$$

**Proof** **of Theorem 1.** Define a Lyapunov function

(14) $$V_{1}\left(s_{xi}\right)=\frac{1}{2}s_{xi}\cdot s_{xi}.$$

Taking the time derivative of $V_{1}\left(s_{xi}\right)$ yields

(15) $$\begin{array}{c} \begin{array}{cc} \frac{V_{1}\left(s_{xi}\right)}{dt}\\ = & s_{xi}\cdot \left({\dot{x}}_{i}-{\dot{g}}_{xi}\right)\\ = & s_{xi}\cdot \left(u_{xi}+d_{xi}-{\dot{g}}_{xi}\right)\\ = & s_{xi}\cdot (-b_{1}\|s_{xi}{\|}^{\beta_{1}}sgn\left(s_{xi}\right)-b_{2}\left\|s_{xi}{\|}^{\beta_{2}}sgn\left(s_{xi}\right)-\rho \cdot sgn\left(s_{xi}\right)+d_{xi}\right)\\ = & -b_{1}\|s_{xi}{\|}^{\beta_{1}+1}-b_{2}\|s_{xi}{\|}^{\beta_{2}+1}-\left(\rho \right\|s_{xi}\left\|-d_{xi}\cdot s_{xi}\right). \end{array} \end{array}$$

Since the input disturbance $d_{xi}\left(t\right)$ is bounded ($\|d_{i}\left(t\right)\|<\rho$), it can be given that $d_{xi}\cdot s_{xi}<\rho \left\|s_{xi}\right\|$. For function (15), it gives

(16) $$\begin{array}{c} \begin{array}{cc} \frac{V_{1}\left(s_{xi}\right)}{dt}< & -b_{1}\|s_{xi}{\|}^{\beta_{1}+1}-b_{2}\|s_{xi}{\|}^{\beta_{2}+1}-\left(\rho \right\|s_{xi}\left\|-\rho \cdot \right\|s_{xi}\left\|\right)\\ < & -b_{1}\left\|s_{xi}{\|}^{\beta_{1}+1}-b_{2}\right\|s_{xi}{\|}^{\beta_{2}+1}\\ < & -{\left\{2{\left(b_{1}\right)}^{\frac{2}{\beta_{1}+1}}V_{1}\left(s_{xi}\right)\right\}}^{\frac{\beta_{1}+1}{2}}-{\left\{2{\left(b_{2}\right)}^{-\frac{2}{\beta_{2}+1}}V_{1}\left(s_{xi}\right)\right\}}^{\frac{\beta_{2}+1}{2}}. \end{array} \end{array}$$

According to the Lemma 2, for the time $t>T_{1}$,

$$T_{1}=\frac{1}{2{\left(b_{1}\right)}^{\frac{2}{\beta_{1}+1}}\left(1-\frac{\beta_{1}+1}{2}\right)}+\frac{1}{2{\left(b_{2}\right)}^{-\frac{2}{\beta_{2}+1}}\left(\frac{\beta_{2}+1}{2}-1\right)},$$

$s_{xi}=0$ is implemented. Similarly, $s_{yi}=0$ can be implemented within a fixed time.

Next, the lower bounds of the ${u_{xi\_}}_{\max},{u_{yi\_}}_{\max}$ are analyzed. When $t<T_{1}$, it has to satisfy

$$\begin{array}{c} \begin{array}{cc} -u_{xi\_\max} & \leq {\dot{g}}_{xi}-b_{1}\|s_{xi}{\|}^{\beta_{1}}sgn\left(s_{xi}\right)\\ & \;\;\;-b_{2}\|s_{xi}{\|}^{\beta_{2}}sgn\left(s_{xi}\right)-\rho \cdot sgn\left(s_{xi}\right)\\ & \leq u_{xi\_\max}, \end{array} \end{array}$$

such that

$$\begin{array}{c} -u_{xi\_\max}-{\dot{g}}_{xi}+\rho \cdot sgn\left(s_{xi}\right)\leq \\ -b_{1}\left\|s_{xi}{\|}^{\beta_{1}}sgn\left(s_{xi}\right)-b_{2}\right\|s_{xi}{\|}^{\beta_{2}}sgn\left(s_{xi}\right)\\ \leq u_{xi\_\max}-{\dot{g}}_{xi}+\rho \cdot sgn\left(s_{xi}\right). \end{array}$$

When $s_{xi}>0$, the maximum value $u_{xi\_\max}$ needs to satisfy

$$-u_{xi\_\max}-{\dot{g}}_{xi}+\rho <0,$$

which can be rewritten as

$$u_{xi\_\max}>-{\dot{g}}_{xi}+\rho .$$

When $s_{xi}<0$, the maximum value $u_{xi\_\max}$ needs to satisfy

$$u_{xi\_\max}-{\dot{g}}_{xi}+\rho \cdot sgn\left(s_{xi}\right)>0,$$

which can be rewritten as

$$u_{xi\_\max}>{\dot{g}}_{xi}-\rho .$$

In conclusion, it gives

$$u_{xi\_\max}>\left\|{\dot{g}}_{xi}\right\|+\rho .$$

Similarly, the maximum value $u_{yi\_\max}$ needs to satisfy

$$u_{yi\_\max}>\left\|{\dot{g}}_{yi}\right\|+\rho .$$

Hence, the maximum values of the control inputs need to be

(17) $$\left[\begin{array}{c} {u_{xi\_}}_{\max}\\ {u_{yi\_}}_{\max} \end{array}\right]\geq \left[\begin{array}{c} \rho +\|{\dot{g}}_{xi}\|\\ \rho +\|{\dot{g}}_{xi}\| \end{array}\right].$$

The above analysis proves that the controller (13) can force states in the robot network to stabilize on the sliding surfaces in a fixed time, and the lower bounds for the maximum values of the control inputs are given.  □

**Remark** **1.** *It can be noted that the values of ${\dot{g}}_{xi},{\dot{g}}_{yi}$ are related to ${\dot{C}}_{i}$ and parameters $k_{1},k_{2},k_{3},k_{4}$. The smaller the values of $k_{1},k_{2},k_{3},k_{4}$, the closer the values of ${\dot{g}}_{xi},{\dot{g}}_{yi}$ are to ${\dot{C}}_{xi},{\dot{C}}_{yi}$. In addition, it can be noted that the larger the values of ${u_{xi\_}}_{\max},{u_{yi\_}}_{\max}$, the larger the coefficients $b_{1},b_{2},b_{3},b_{4},\beta_{2},\beta_{4}$ can be, the smaller the coefficients $\beta_{1},\beta_{3}$ can be and the smaller $T_{1}$ can be.*

**Theorem** **2.** *When the robot’s state reaches the sliding surface (11), the controller (13) can drive the position $p_{i}$ of robot i(i∈V), track the Voronoi centroid $C_{i}$ within a fixed time $T_{2}$ and the time optimal coverage effect is achieved, where*

$$T_{2}=T_{1}+\frac{1}{2{\left(k_{1}\right)}^{\frac{2}{\alpha_{1}+1}}\left(1-\frac{\alpha_{1}+1}{2}\right)}+\frac{1}{2{\left(k_{2}\right)}^{-\frac{2}{\alpha_{2}+1}}\left(\frac{\alpha_{2}+1}{2}-1\right)}.$$

**Proof** **of Theorem 2.** Define a positive definite Lyapunov function

(18) $$V_{2}\left(e_{xi}\right)=\frac{1}{2}\left(x_{i}-C_{xi}\right)\cdot \left(x_{i}-{C_{x}}_{i}\right).$$

Taking the time derivative of $V_{2}\left(e_{xi}\right)$ yields

(19) $$\begin{array}{c} \begin{array}{cc} \frac{V_{2}\left(e_{xi}\right)}{dt}= & \left(x_{i}-C_{xi}\right)\cdot \left({\dot{x}}_{i}-{\dot{C}}_{xi}\right)\\ = & \left(x_{i}-C_{xi}\right)\cdot \left(u_{xi}+d_{xi}-{\dot{C}}_{xi}\right). \end{array} \end{array}$$

According to Theorem 1, when $t>T_{1}$, $s_{xi}=0,s_{yi}=0$ and $\dot{x}={\dot{g}}_{xi}$ can be obtained. For the function (19), one has

(20) $$\begin{array}{c} \begin{array}{cc} \; & {\dot{V}}_{2}\left(e_{xi}\right)\\ \; & =e_{xi}\cdot (-k_{1}\|e_{xi}{\|}^{\alpha_{1}}sign\left(e_{xi}\right)-k_{2}\left\|e_{xi}{\|}^{\alpha_{2}}sign\left(e_{xi}\right)\right)\\ \; & =-(k_{1}\|e_{xi}{\|}^{\alpha_{1}+1}+k_{2}\|e_{xi}{\|}^{\alpha_{2}+1})\\ \; & =-({\left\{2{\left(k_{1}\right)}^{\frac{2}{\alpha_{1}+1}}\frac{1}{2}{e^{2}}_{xi}\right\}}^{\frac{\alpha_{1}+1}{2}}+{\left\{2{\left(k_{2}\right)}^{\frac{2}{\alpha_{2}+1}}\frac{1}{2}{e^{2}}_{xi}\right\}}^{\frac{\alpha_{2}+1}{2}})\\ \; & =-{\left\{2{\left(k_{1}\right)}^{\frac{2}{\alpha_{1}+1}}V_{2}\left(e_{xi}\right)\right\}}^{\frac{\alpha_{1}+1}{2}}-{\left\{2{\left(k_{2}\right)}^{\frac{2}{\alpha_{2}+1}}V_{2}\left(e_{xi}\right)\right\}}^{\frac{\alpha_{2}+1}{2}}. \end{array} \end{array}$$

According to Lemma 2, the position error $e_{xi}$ can be stabilized to 0 ($e_{xi}=0$) within the fixed time $T_{2}$, where

$$T_{2}=T_{1}+\frac{1}{2{\left(k_{1}\right)}^{\frac{2}{\alpha_{1}+1}}\left(1-\frac{\alpha_{1}+1}{2}\right)}+\frac{1}{2{\left(k_{2}\right)}^{-\frac{2}{\alpha_{2}+1}}\left(\frac{\alpha_{2}+1}{2}-1\right)}.$$

The quantify $u_{xi\_\max}$ needs to satisfy

$$\begin{array}{c} \begin{array}{cc} & -u_{xi\_\max}\leq {\dot{g}}_{xi}-b_{1}\|s_{xi}{\|}^{\beta_{1}}sgn\left(s_{xi}\right)\\ & -b_{2}\|s_{xi}{\|}^{\beta_{2}}sgn\left(s_{xi}\right)-\rho (\|s_{xi}{\|}^{\beta_{1}}+\left\|s_{xi}{\|}^{\beta_{2}}\right)sgn\left(s_{xi}\right)\\ & \;\;\;\;\;\;\;\;\;\;\;\;\;\leq u_{xi\_\max}. \end{array} \end{array}.$$

When $t>T_{1}$, one has $s_{xi}=0$, and

$$\begin{array}{c} -b_{1}\|s_{xi}{\|}^{\beta_{1}}sgn\left(s_{xi}\right)-b_{2}\|s_{xi}{\|}^{\beta_{2}}sgn\left(s_{xi}\right)-\rho (\|s_{xi}{\|}^{\beta_{1}}+\left\|s_{xi}{\|}^{\beta_{2}}\right)sgn\left(s_{xi}\right)=0. \end{array}$$

Therefore, the quantity $u_{xi\_\max}$ needs to satisfy

$$-u_{xi\_\max}\leq {\dot{g}}_{xi}\leq u_{xi\_\max}.$$

Substituting (12) gives

$$\begin{array}{c} -u_{xi\_\max}\leq {\dot{C}}_{xi}-k_{1}\left\|e_{xi}{\|}^{\alpha_{1}}sgn\left(e_{xi}\right)-k_{2}\right\|e_{xi}{\|}^{\alpha_{2}}sgn\left(e_{xi}\right)\leq u_{xi\_\max}, \end{array}$$

since the parameters $k_{1},k_{2}\left(k_{1},k_{2}>0\right)$ can be arbitrarily small positive numbers, such that

(21) $$u_{xi\_\max}>\left\|{\dot{C}}_{xi}\right\|.$$

In Theorem 1, the lower bound of the control input $u_{xi\_\max}$ is given in (17), which has already satisfied the condition (21). Similarly, $e_{yi}=0$ can be obtained within a fixed time $T_{2}$. Therefore, the robot network can achieve the optimal position configuration in a fixed time, and the time optimal coverage effect for the task area is achieved.  □

**Remark** **2.** *If the quantities ${u_{xi\_}}_{\max},{u_{yi\_}}_{\max}$ are large enough, the parameters $k_{1},k_{2},k_{3},k_{4},\alpha_{2},\alpha_{4}$ can be designed for larger values and the parameters $\alpha_{1},\alpha_{3}$ can be designed for smaller values. Then, the fixed time $T_{2}$ can be smaller, and the robot i(i∈V) can track the Voronoi centroid faster.*

## 5. Simulation Examples

Several simulation experiments were carried out to verify the proposed T-OCC method. Consider a 100 m × 100 m convex 2-D area, the robot network composed of four robots with maximum control inputs of 8 m/s, 10 m/s, 12 m/s and 9 m/s performed the area coverage task cooperatively. There were two important movable objects in the task area, and their motion trajectory was as follows:

$$\begin{array}{c} \begin{array}{cc} x_{1}=30-10\sin\left(t/15\right),\; & y_{1}=30+10\cos\left(t/15\right),\\ x_{2}=90-0.5t,\; & y_{2}=90-0.5t. \end{array} \end{array}$$

The contribution function $\phi_{j}\left(q,t\right)$ of the object *j* to the risk density is given as

$$\phi_{1}\left(q,t\right)=5e^{-\frac{\|q-m_{1}\left(t\right){\|}^{2}}{200}},\;\phi_{2}\left(q,t\right)=3e^{-\frac{\|q-m_{2}\left(t\right){\|}^{2}}{200}},$$

**Figure 3:**
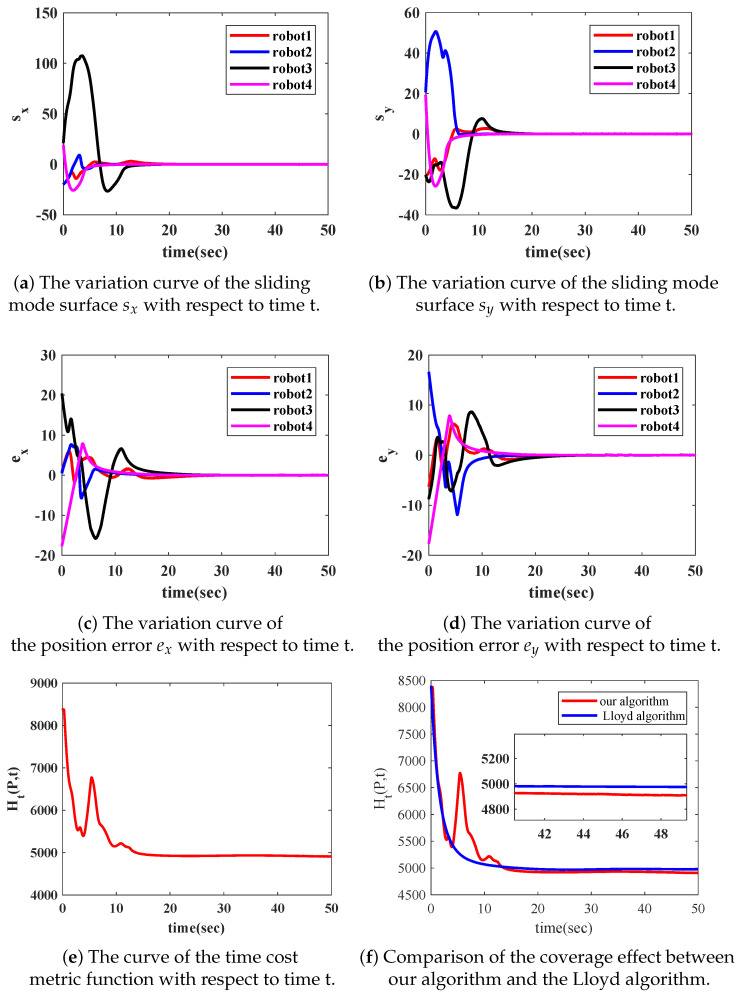
The variation curves of $s_{x}$, $s_{y}$, $e_{x}$ and $e_{y}$ of the four robots with respect to time, the variation curves of metric function $H_{t}\left(P,t\right)$ and the comparison of optimization effects with the classic Lloyd algorithm.

The input disturbance is given as $d_{xi}=1*\sin\left(t\right),d_{yi}=1*\cos\left(t\right)$. When the parameters of the designed controller were $k_{1}=k_{2}=k_{3}=k_{4}=0.2$, $\alpha_{1}=0.8,\alpha_{2}=2$, $b_{1}=b_{2}=b_{3}=b_{4}=0.2$ and $\beta_{1}=0.5,\beta_{2}=2$, the variation curves of states $s_{x}$, $s_{y}$ are shown in Figure 3a,b, and the position errors $e_{x}$, $e_{y}$ are shown in Figure 3c,d. It can be noted that the sliding mode surfaces $s_{x}$ and $s_{y}$ stabilized to 0 within 20 s, and the position errors $e_{x}$ and $e_{y}$ stabilized to 0 within 30 s.

**Figure 4:**
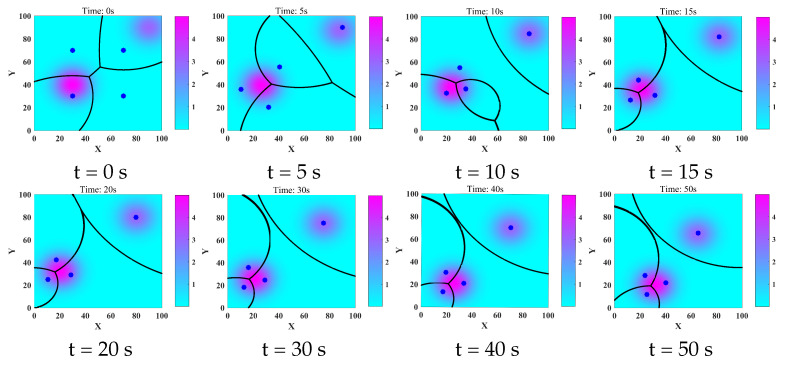
The coverage evolution process of the robot network to the task area, where the blue point “•” represents the position of the robot, and different colors in the area represent different risk degrees.

We compared the proposed control algorithm (13) with the classical Lloyd algorithm [9,26]. The time evolution of the TCMF $H_{t}\left(P,t\right)$ is shown in Figure 3e. In Lloyd’s algorithm, the control proportionality coefficient was set as 0.4, so that the two algorithms made the decrease rate of the metric function almost equal to the initial time. Figure 3f shows the comparison of the two algorithms. It could be noted that the control algorithm (13) could make the metric function smaller, therefore, the control algorithm had a better effect. The coverage evolution process of the robot network is shown in Figure 4. The distribution of the robot network was random at the initial time. Then, the robot network moved to the optimal position configuration and maintained the optimal coverage effect, despite several important objects in the area that were constantly moving.

## 6. Conclusions

In this work, we studied the ACCP for a robot network in a dynamic environment considering the time cost. The most important findings are listed as follows:

1. When it is necessary to respond quickly to accidents, the coverage time cost is introduced to measure the coverage effect of the robot network on the task area;
2. Based on the TCMF, a fixed-time robust controller was designed to drive the robot network to achieve the minimum coverage time cost considering input disturbances;
3. The conditions that the maximum value of the control inputs should satisfy were obtained.

Collision avoidance in the coverage control will be the subject of future research.

## Data Availability

Not applicable.
